# Overall quality of life impact on candidates for septorhinoplasty according to the World Health Organization quality of life brief questionnaire (WHOQOL-Brief)

**DOI:** 10.1016/j.bjorl.2020.07.015

**Published:** 2020-09-14

**Authors:** Paula de Oliveira Oppermann, Luísi Rabaioli, Cassia Feijó, Natália Paseto Pilati, Emily Nicole Hrisomalos, Raphaella de Oliveira Migliavacca, Michelle Lavinsky-Wolff

**Affiliations:** a Universidade Federal do Rio Grande do Sul (UFRGS), Pós-Graduação em Pneumologia, Porto Alegre, RS, Brazil; b Hospital de Clínicas de Porto Alegre, Serviço de Otorrinolaringologia e Cabeça e Pescoço, Porto Alegre, RS, Brazil; c Universidade Federal do Rio Grande do Sul (UFRGS), Faculdade de Medicina, Porto Alegre, RS, Brazil; d Turkle and Associate Cosmetic and Reconstructive Surgery, Carmel, United States

**Keywords:** Quality of life, Septorhinoplasty, Nasal obstruction, WHOQOL-Brief

## Abstract

**Introduction:**

Quality of life has been an increasingly reference measure in whole health impact of diseases and in septorhinoplasty evaluation as well. It is known that the decision for this elective surgical procedure requires the subjective perception of patients’ complaints about their own health and life stage in association with the surgeon’s aesthetic and functional perspective of each case.

**Objective:**

To define the quality of life of candidates for septorhinoplasty using the World Health Organization quality of life questionnaire, WHOQOL-Brief, and the prevalence of other independent variables for this population.

**Methods:**

A cross-sectional study using a sample of candidates for septorhinoplasty was performed. All patients responded to the WHOQOL-Brief during the pre-operative period. A normative population quality of life study was the reference for the sample size and means.

**Results:**

A total of 302 patients were included among the 322 eligible patients. Twenty patients did not complete the questionnaire correctly and were excluded from the study. The sample consisted of patients aged between 15 and 78 years (34.7 ± 14 years): the most majority were Caucasian and female. Among this group, 88.1% declared symptoms of nasal obstruction and 77.4% complained of sleeping problems. It was seen that 10.9% patients chose the surgery primarily for aesthetic improvement; 37.1% chose it mainly because of functional symptoms and 52% chose it for both functional and aesthetic reasons. The physical health domain’s mean was 62.2 ± 17), which is a higher mean compared to the references’ standard one (μ = 58.9 ± 10.5, *p* = 0.002). The social relationship domain mean was 70.8 ± 18.1; that is a lower mean then general population’s one (μ = 76.2 ± 18.8, *p* < 0.001). The psychological and the environment domain means revealed no difference when comparing the sample to the norm (μ = 65.3 ± 15.1 vs. μ = 65.9 ± 10.8, *p* = 0.530 and μ = 60.3 ± 13.1 vs. μ = 59.9 ± 14.9, *p* = 0.667).

**Conclusion:**

The WHOQOL-Brief questionnaire proved an accurate instrument to cross-check different populations in quality of life outcomes. The study provides good evidence of lower quality of life in social relations domain and high prevalence of nasal obstruction and sleeping symptoms in candidates for septorhinoplasty. This study contributes to recent literature with relevant data supporting a more integrative evaluation in this population in the preoperative period. The results may also encourage a multidisciplinary approach for chronic symptoms when associated with nasal obstruction, sleep disorders and aesthetic complaints.

## Introduction

Aesthetic nasal surgery is currently one of the most common surgical procedures worldwide and, therefore, its benefits and techniques are widely discussed. Patient satisfaction and gain in quality of life (QoL) are the main parameters that define the success of this surgery.^1^ It is known that this satisfaction may be influenced by many aspects involving aesthetic and functional outcomes in the same patient. Nasal complaints related to its function do not present a directly proportional relation with quantitative respiratory area exams. Thus, it is already established that the patient's subjective symptomology is the main parameter that defines diagnosis of nasal obstruction.^2^, ^3^

Research involving self-evaluation of specific aesthetic and/or functional symptom questionnaires are primarily using the Nasal Outcome Symptom Evaluation (NOSE-p) and the Rhinoplasty Outcome Evaluation (ROE) questionnaires. In the currently available literature, studies generally advocate an improvement in a specific QoL related to nasal obstruction, when using these tools. However, the literature contains a gap in the sense that as yet it does not have an efficient questionnaire to evaluate the patient under more than one dimension of life.^1^ New studies including the general QoL questionnaires have emerged in association with NOSE-p in the evaluation of septorhinoplasty results.^4^ In addition, widespread use of these general QoL questionnaires provide for obtaining general QoL data of patients with different chronic diseases and normative data regarding different sociocultural contexts.^5^, ^6^

This study employed the questionnaire of the World Health Organization Brief version (WHOQOL-Brief), that is an instrument with growing importance in recent literature. The WHOQOL-Brief has 26 questions that cover four domains: physical health, psychological, social relationship, and personal environment. This questionnaire is validated for the Portuguese language and has been used in a relevant study published in 2011 that stablished the QoL general average of the local population in south Brazil.^7^ This previous research studied a local normative sample of 751 individuals who were evaluated from each score of the WHOQOL-Brief domains.^8^ There are further new studies using QoL questionnaires, and some of them advocate in association of emotional and psychological domain lower results for patients who desire septorhinoplasty.^9^, ^10^ (I am unable to make any sense out of this sentence, and it needs revision) Two clinical trials with this same scope have been published using the WHOQOL-Brief to evaluate patients' general QoL after septorhinoplasty in association with nasal turbinate procedures (cauterization/endoscopic partial turbinectomy inferior). They showed no difference in the QoL scores before and after turbinate intervention.^3^, ^11^

The current study aims to define the mean scores for general QoL in patients that are candidates for septorhinoplasty using the WHOQOL-Brief tool and to compare it with local population normative data and independent factors that influence general QoL.

## Methods

A cross-sectional study was carried out with a population in south Brazil. Data were collected during the period of March 2011 and March 2017 through SPPS database, version 20.0. The sample consisted of individuals who met the inclusion criteria: they were referred to the outpatient clinic for septorhinoplasty for both functional and aesthetic nasal complaints; they are willing to participate of the study; and they have consented through an Informed Consent Term. Exclusion criteria included being under 16 years of age, being illiterate, having technical contraindication to the procedure and/or having indications for another associated surgical procedure such as blepharoplasty, otoplasty or sinus surgery.

To perform the sample size, we considered the normative study, which presented a mean value of 58.9 and a standard deviation of 17. An average score of five points was expected, and it was necessary to evaluate 182 patients. A power of 80% was considered, and the WinPeppi software was used to perform a level of significance of 0.05.

All patients included in the study consented to participate by signing an informed consent form. The study was approved by the Hospital's Ethics Committee Ethics Committee, under number CAAE 62058016.8.0000.5327. Once included in the study, the patients were instructed to respond to the following quality of life questionnaires:

WHOQOL-Brief: A research questionnaire with 26 questions regarding the overall QoL. The issues are divided into categories according to their relevance in four domains: physical health, psychological, social, and personal environment. The results of each domain have been calculated using the SPSS software with the World Health Organization (WHO) formula, based on the answers to each question to provide the scores of the four different domains. Each item is marked 1–5 and is transformed into a linear scale between 1 and 100, the latter being the most favorable.^8^

We used normative data from the WHOQOL-Brief in the general population of Porto Alegre as reference for comparative analysis with the results of the sample of the patients that were candidates for rhinoseptoplasty.^7^

ROE: It is a questionnaire with six questions covering three QoL domains: physical, mental/emotional and social. As defined by the authors, each question is presented with a four-point Likert Scale. The total score is divided by 24 and multiplied by 100 to reach the final score, which can range from 0 to 100. A score of 100 means extreme satisfaction, while score 0 indicates the highest degree of dissatisfaction.^2^

NOSE: It is composed of five items related to the severity of nasal obstruction in the last month: nasal congestion or sensation of a full nose; blockage or nasal obstruction; difficulty of breathing through the nose; difficulty of sleeping; and inability to breathe enough through the nose during exercise or exertion. The five items are scored on a five-point Likert Scale. A score from 0 to 100 is generated by multiplying the total value obtained by five. The higher the score, the greater is the intensity of the problem related to nasal obstruction.^12^

The categorical variables were described in the form of absolute and relative frequencies while the continuous symmetrical distribution variables were described in the form of mean and standard deviation. The relationship between continuous variables was verified with the Pearson Correlation Coefficient. To compare the QoL scores with the scores of Porto Alegre normative data, *t*-test Student was used. To verify factors associated with QoL in every domain, the simple and the later multiple linear regression analysis was used. A significance level of 0.05 was considered for all analyses and the software that was used is SPSS 20.0.

## Results

**Figure 1:**
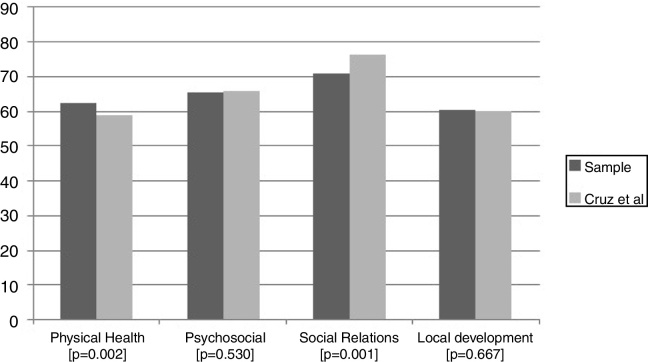
Graphic demonstration of the results from comparative analysis of the sample mean in each domain of WHOQOL-Brief and means obtained from the normative study data (Cruz et al.)^8^ using *t*-Student test. The difference was considered statistically significant when *p* < 0.05.

**Figure 2:**
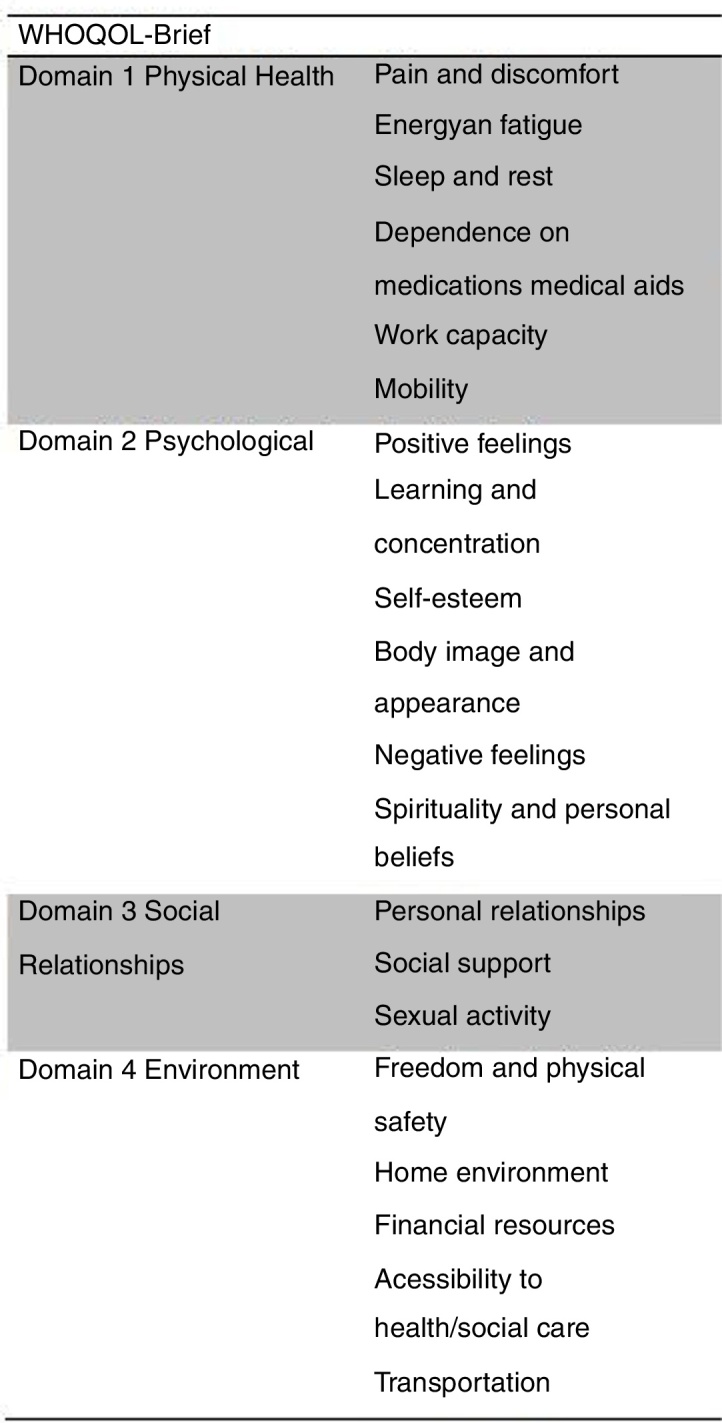
Description of domains adapted from World Health Organization Quality of Life Brief questionnaire—www.who.int/mental_health/media/en/76.pdf.

A total of 302 patients were included among the 322 eligible patients. Twenty patients did not complete the questionnaire accordingly and were excluded from the study. The sample consisted of patients aged between 15 and 78 years (34.7 ± 14 years): most were Caucasian and female as shown in Table 1. Among these candidates for rhinoseptoplasty, 88.1% declared to have symptoms of nasal obstruction and 77.4% complained of sleeping difficulties. It was seen that 10.9% patients chose this procedure primarily for aesthetic improvement; 37.1% chose it mainly because of functional symptoms and 52% chose it for both functional and aesthetical intention equally. Regarding the education level, the majority of the sample (45.4%) reported between 9 and 11 years of schooling and 32.8% had less than 8 years of schooling. Table 1 shows other characteristics of the sample. Fig. 1 demonstrates the behavior of the WHOQOL-Brief in their domains and Fig. 2 shows the qualitative measures in each domain of the questionnaire. The mean value of the physical health domain was 62.2 (±SD = 17), being significantly higher than the reference used in the Porto Alegre standard (μ = 58.9, SD = 10.5, *p* = 0.002). Relating to the social relations domain, the mean score was 70.8 (±SD = 18.1), which was different than that of the norm (μ = 76.2, SD = 18.8, *p* < 0.001). In the psychological and personal environment domains, there was no significant difference between the sample and the normative (μ = 65.3 ± 15.1 vs. μ = 65.9 ± 10.8, *p* = 0.530 and μ = 60.3 ± 13.1 vs. μ = 59.9 ± 14.9, *p* = 0.667, respectively). Factors associated with each domain were unadjusted, univariate, and adjusted for age, sex, and schooling. Factors that presented a level of significance lower than 0.1 were considered for multivariate adjustment. The following variables were included in the multivariable analysis: sex, age, schooling, associated comorbidities, and nasal symptoms when sleeping.

**Table 1:** Main results from sample frequencies and mean analyses.

	n = 302 (%)
Female	170 (56.3)
Mean Age (SD)	34.7 (14)
Caucasian	268 (88.7)
Education	
Less than 8 years	99 (32.8)
9 to 11 years	137 (45.4)
12 years or more	66 (21.8)
Morbidities present	130 (43.0)
Objective	
Functional	112 (37.1)
Aesthetic alone	33 (10.9)
Both	157 (52.0)
Nasal surgery before	50 (16.6)
Nasal obstruction	266 (88.1)
Sleep symptoms	222 (77.4)
Previous nasal trauma	106 (35.2)
WHOQOL-Brief	
Physical health, mean (SD)	62.2 (17.0)
Psychosocial, mean (SD)	65.3 (15.1)
Social relationships, mean (SD)	70.8 (18.1)
Environment, mean (SD)	60.3 (13.1)
General, mean (DP)	61.3 (19.8)

Table 2 shows that in the physical health field, the presence of some comorbidity or the complaint of symptoms during sleep was significantly associated with a score decrease of −7.4 or −8.9, respectively. In relation to the psychological domain, the presenting comorbidity is associated with a drop of four points in the mean QoL score. In the psychological, the social relations and the personal environment domains, patients with 12 years of schooling or more had higher mean values ​​than patients with up to eight years of schooling. It was demonstrated that in addition to chronic diseases, the age factor, the presence of nasal symptoms during sleep and the presence of nasal obstruction were related to a drop in QoL in the General Physical Health domain (*p* < 0.05). The ROE questionnaire has presented an average value of 31.63 ± 16.5 and the NOSE-p an average of 67.8 ± 27. Pearson's linear correlation analysis showed that the strongest relationship found was −0.33 between NOSE-p and the WHOQOL-Brief physical health domain. Although the psychological and the social relations domains have a linear Pearson correlation, it is different from 0 to ROE, the magnitude of the correlation is weak as seen in Table 3.

**Table 2:** Description of variables included in the multivariable analysis: sex, age, schooling, associated morbidities and nasal symptoms present during sleep as independent factors for results in each domain of WHOQOL-brief. The coefficient was adjusted in each domain.

	Regression Coefficient			
	Physical health	Psychosocial	Social relationship	Environment
Female	1.26 (−2.51;5.04)	−2.72 (−6.28;0.83)	1.51 (−2.63;5.66)	1.35 (−1.65;4.34)
Age	−0.16 (−0.3;−0.02)^a^	0.03 (−0.16;0.11)	−0.049 (−0.20;0.10)	−0.05 (−0.16;0.06)
Education				
Less than 8 years	−	−	−	−
9 to 11 years	2.08 (−2.37;6.52)	1.42 (−2.77;5.61)	3.73 (−1.16;8.61)	0.98 (−2.55;4.52)
12 years or more	5.04 (−0.19;10.23)	6.23 (1.31;11.2)^a^	9.23 (3.44;15.02)^a^	6.09 (1.9;10.3)^a^
Morbidities	−7,4 (−11.3;−3.5)^a^	−4 (−7.62;−0.36)^a^	−	−
Sleeping symptoms	−8.89 (−13.3;−4.5)^a^	−	−	−3.30 (−6.85;0.25)

a *p* < 0.005.

**Table 3:** WHOQOL-brief Linear Domains Interface with NOSE-p and ROE results for Pearson’s Correlation.

WHOQOL-Brief	NOSE-p	ROE
Physical Health	−0.333^a^	0.033
Psychosocial	0.035	0.120^a^
Social relationships	0.068	0.114^a^
Environment	−0.016	0.098
General	−0.217^a^	0.126

a Pearson’s Correlation *p* < 0.05.

## Discussion

In the pertinennt literature a demand for an efficient questionnaire to evaluate the patient under more than one dimension of his life is suggested.^1^ This study used the Pearson Correlation tool to compare the trend of the different questionnaires results and, even though weak, it has an inverse behavior of the NOSE-p questionnaire with the WHOQOL-Brief. While using the correlation tool with different questionnaires, we wanted to provide a model for future researches to evaluate more than one dimension of the individual’s problems, even when there is no questionnaire available to cover all the complaints simultaneously.

This study describes overall QoL scores of patients who are candidates for septorhinoplasty using the WHOQOL-Brief questionnaire that has been used in different areas. It particularly presents a good capacity to identify patients from different populations with emotional symptoms.^12^ It was demonstrated that the QoL in patients who are candidates for septorhinoplasty, as measured by the WHOQOL-Brief, was lower for the domain of social relationships when compared with the results obtained in the reference study of the population of Porto Alegre used for the sample size and means.^8^ Another cross-sectional study recently demonstrated a similar tendency of that population in presenting preoperative and early postoperative symptoms of stress and anxiety, as well as worse self-esteem and low quality of sexual life when other quality of life questionnaires is used such as Questionnaire F9 Life Satisfaction (FLZ), Rosemberg Self Steem Questionary (RSES), and Glasgow Benefit Inventory (GBI), whose data were also compared with normative data of the local population.^13^ The current study data reaffirms the ability of QoL questionnaires to translate aspects of the individual's emotional self-perception and their social context also when using WHOQOL-Brief.

Regarding the physical health domain, the patients in the sample had a higher mean of general QoL, as shown in Fig. 1, than the average of the same domain, according to data from the normative population study.^8^ This data can be explained by the fact that people tend to undergo elective procedures when they are in a good health, something that may influence the way they deal with surgery and their expectations therefrom. It was found that presenting chronic diseases, advanced age and nasal symptoms during sleep were related to a significant drop in QoL in this domain for this population. When analyzing other studies of QoL that had used WHOQOL-Brief, it was found that subgroups of patients with chronic diseases have generally worse QoL scores.^7^ The mean scores of this sample in physical domain is lower than the chronic disease study means (62.2 ± SD = 17 vs. 72.9).^6^ These associations suggest further research to compare the impact of any other chronic diseases using QoL scores as well. Another recently systematic review suggested the relevance of the patient-reported outcome measures questionnaires in chronic nasal obstruction symptom evaluation, as it showed a significant improvement of the results after surgery in the first postoperative year.^4^ This study’s prevalence of functional complaints prove it to be an important motivation for septorhinoplasty and reaffirm the importance of exploring nasal obstruction complaints and sleeping symptoms in the preoperative evaluation.

Relating to the psychological and personal environment domains, the study found no difference in QoL scores when compared with the normative data.^8^ A similarity of our sample to the reference data can be expected, since both present similar population characteristics as the majority of the female gender, average age between 30–44 years old, and a predominance of patients with higher education, since the questionnaire requires literacy to be completed.

A current research has recently demonstrated that septorhinoplasty positively influence patients’ quality of life after surgery, but there is still need to specify the groups which would potentially benefit the most from this procedure.^14^ The present study has specified some characteristics of this population that can take us to a better comprehension of this groups and the situation of life during which they chose this surgery.

The WHOQOL-Brief questionnaire presented a good ability to assess the QoL of patients who are candidates for septorhinoplasty and to discriminate factors associated with worse or better scores. These factors have been influential in the individual's perception of their clinical condition, and therefore, it is understood that the investigation of the preoperative history may help to identify conditions that impact the patient's complaints. The study also brings an understanding of the context in which the individual is living.

## Conclusion

The study provides good evidence of a lower quality of life in the social relations domain in candidates for septorhinoplasty when compared with the general population using the WHOQOL-Brief questionnaire. These results contribute to the discussion about the importance of a subjective and integrative evaluation that can set expectations between surgeons and patients’ satisfaction before and after surgical procedures. It reassures the importance of an individualized decision for each situation and encourage a multidisciplinary approach when chronic symptoms as nasal obstruction and aesthetic complaints exist. The WHOQOL-Brief questionnaire proved to be an accurate instrument for the preoperative evaluation of septorhinoplasty candidates and promises a potential for application in future studies that evaluates the impact of this procedure on the general quality of life of these patients.

## Funding

Academical student support was provided by Coordenação de Aperfeiçoamento de Pessoal de Nível Superior (CAPES). MEC - Fundação Coordenação Aperfeiçoamento de Pessoal de Nível Superior 00.889.834/0001-08 Bolsa de Pos-Graduação.

## Conflicts of interest

The authors declare no conflicts of interest.
